# Supplementary material for: Scavenger receptor-A is a biomarker and effector of rheumatoid arthritis: A large-scale multicenter study
Source: Nat Commun. 2020 Apr 20;11:1911. doi: 10.1038/s41467-020-15700-3 (PMC7171100; doi:10.1038/s41467-020-15700-3)
Supplement: Supplementary file 1 — Supplementary Information [file 41467_2020_15700_MOESM1_ESM.pdf]

Supplementary Information

"Large-scale Multicenter Study Reveals Scavenger Receptor-A as a Novel Biomarker and Exacerbator of Rheumatoid Arthritis"

Hu et al.

Supplementary Figure 1

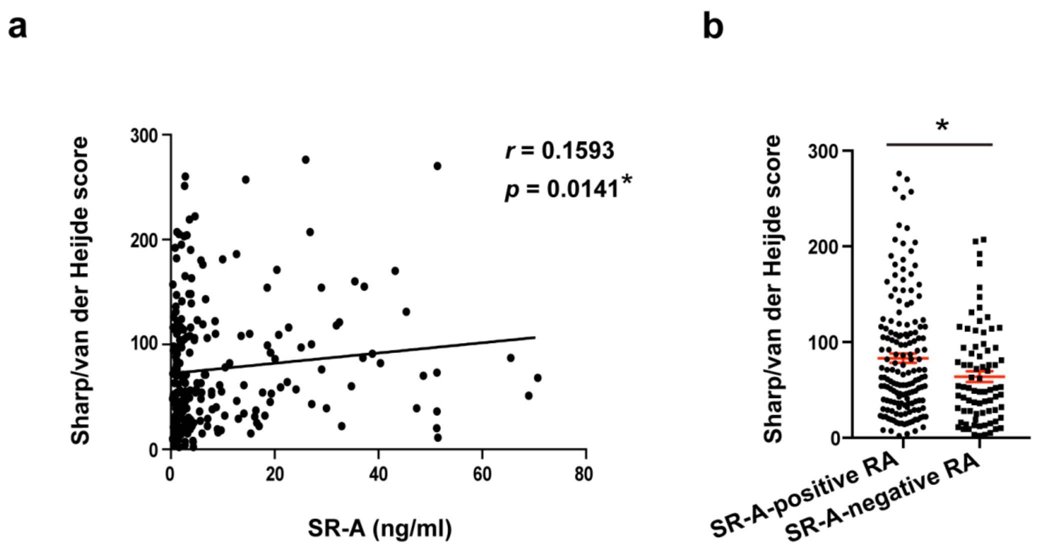

Supplementary Fig. 1 sSR-A levels are modestly correlated with RA patient radiographic damage. (**a**) The radiographic damage in RA patients (n = 237) was assessed by the Sharp/van der Heijde score (SHS), and their correlation with sSR-A levels was further analyzed (*p = 0.0141). (**b**) RA patients were divided into sSR-A-positive (n = 159) and sSR-A-negative (n = 78) groups by the cut-off value, then the corresponding SHS was compared (*p = 0.0201). Red horizontal lines: means; error bars: SEMs**.** *p < 0.05 (two-tailed Spearman's rank correlation test (**a**) or two-tailed Mann-Whitney U test (**b**)). Source data are provided as a Source Data file.

Supplementary Figure 2

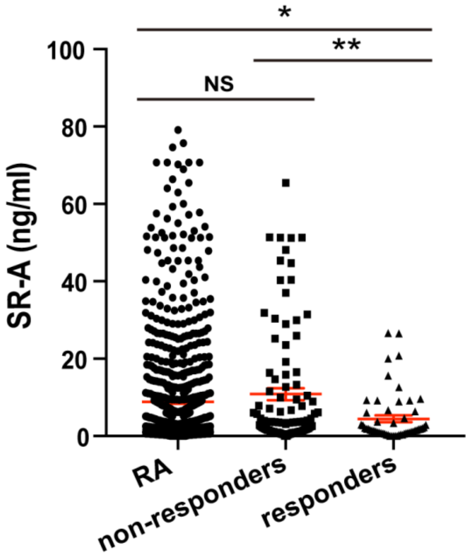

Supplementary Fig. 2 The levels of sSR-A are decreased in the responders but not in the non-responders of RA patients after therapy. The serum sSR-A levels in 100 non-responders (DAS28 > 5.1) and 54 responders (DAS28 < 2.6) of RA patients after therapy were detected by ELISA, and were further compared with the average RA patient levels as tested in Figure 2 (n = 896). Red horizontal lines: means; error bars: SEMs**.** **p* = 0.0159 (< 0.05), ***p* = 0.0083 (< 0.01), NS, not significant (Kruskal–Wallis test followed by Dunn's posttest for multiple comparisons). Source data are provided as a Source Data file.

Supplementary Figure 3

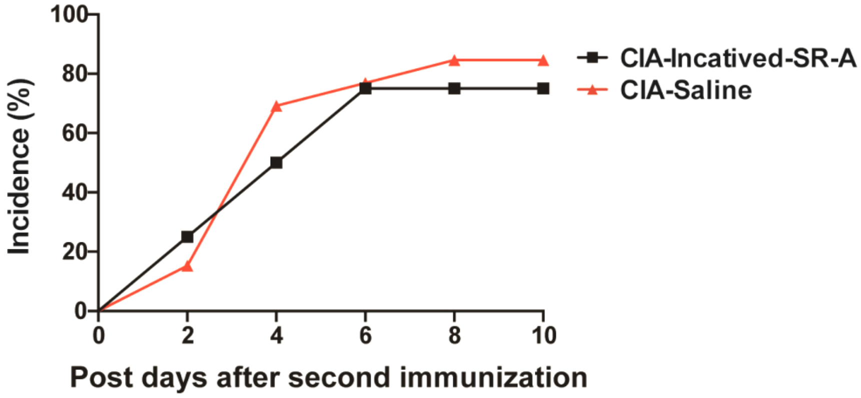

Supplementary Fig. 3 Boiled SR-A protein fails to promote arthritic progression in mice. DBA/1 mice were intravenously injected with boiled recombinant SR-A protein (2 μg/mouse) or saline every two days starting from two days before boosting immunization for a total of 5 times. The arthritis incidence was recorded (CIA-Inactivated SR-A, n = 4; CIA-Saline, n = 5). Data are representative of three independent experiments. Source data are provided as a Source Data file.

Supplementary Figure 4

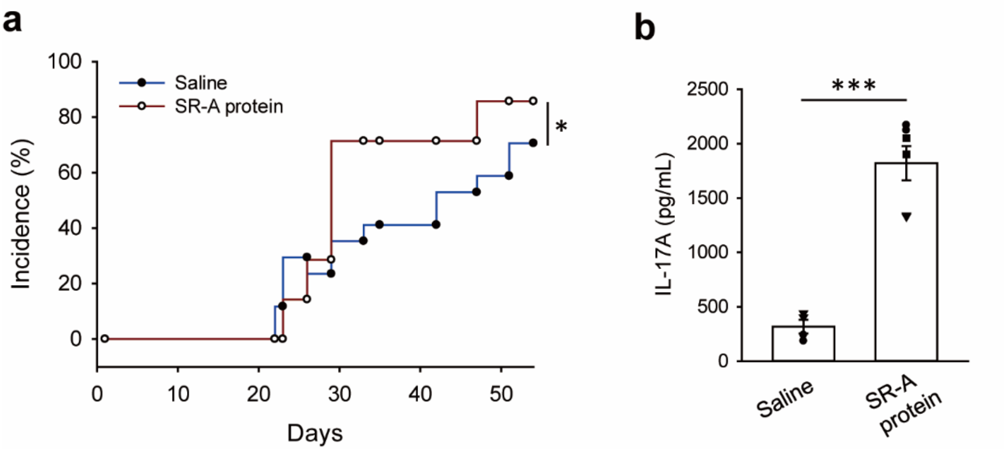

Supplementary Fig. 4 High dose of recombinant SR-A protein exacerbates arthritis in mice. Male DBA/1J mice were immunized with bovine collagen II emulsified in CFA at day 1 and boosted with collagen II emulsified in IFA at day 21. SR-A protein (n = 7, 30 μg/mouse) or saline (n = 17) were administrated every other day for 5 doses starting from day 19. (**a**) Arthritis incidence was followed (**p* = 0.048). (**b**) Three weeks after last injection of SR-A protein, lymph node cells from saline (n = 2) or SR-A protein (n = 3) treated mice were stimulated with collagen II for 48 h. Level of IL-17A in the culture medium was assayed by ELISA in duplicate (****p* = 7.5878E-5). Data are presented as mean ± SEM. Results are representative of three independent experiments. **p* < 0.05, ****p* < 0.001 (LogRank test (**a**) or two-tailed Student's *t* test (**b**)). Source data are provided as a Source Data file.

Supplementary Figure 5

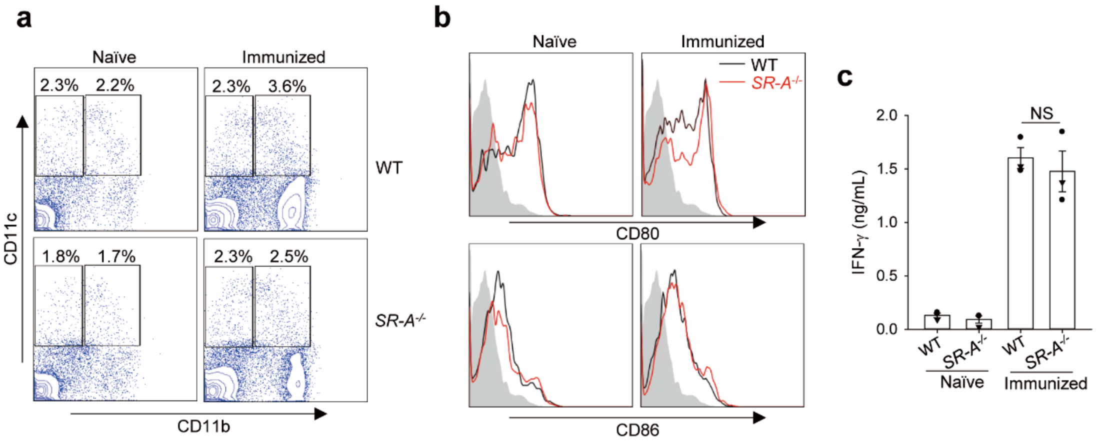

Supplementary Fig. 5 Absence of *SR-A* does not alter dendritic cell function during collagen immunization. WT (n = 3 per group) and *SR-A*$^{-/-}$ (n = 3 per group) mice were immunized with 200 µg collagen II emulsified in CFA or not to induce CIA. (**a** and **b**) Frequency and activation of CD11b^{+}CD11c^{+} dendritic cells in the draining lymph nodes was assayed by flow cytometry one week after immunization. (**c**) Lymph node cells were stimulated with collagen II (50 µg/mL) for 2 days. IFN-γ levels in the culture medium were determined by ELISA. Data are presented as mean ± SEM. Results are representative of three independent experiments. NS, not significant (two-tailed Student's *t* test). Source data are provided as a Source Data file.

Supplementary Figure 6

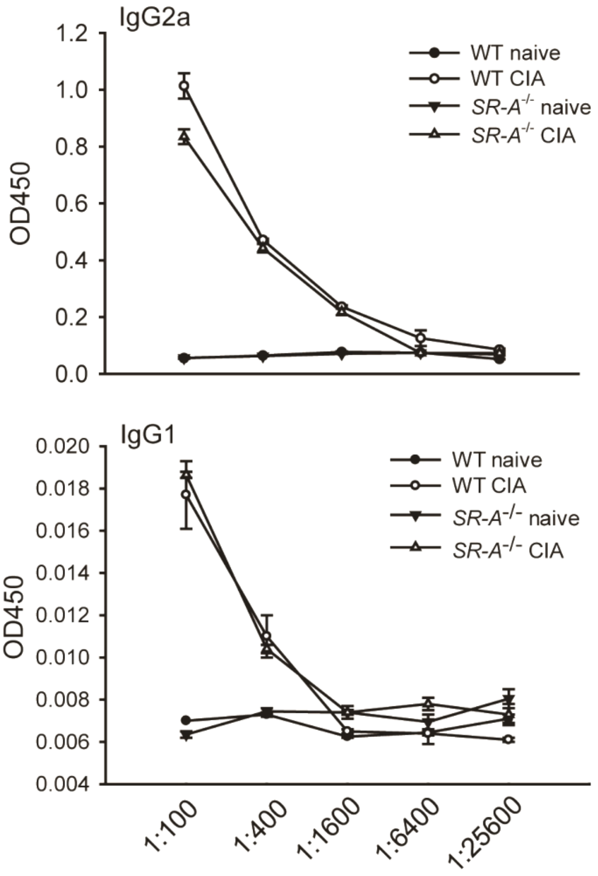

Supplementary Fig. 6 Absence of *SR-A* does not affect an anti-collagen humoral response. WT (n = 3 per group) and *SR-A*$^{-/-}$ (n = 3 per group) mice were immunized with 200 µg collagen II emulsified in CFA or not to induce CIA. Anti-collagen autoantibodies in serum were assayed six weeks post immunization. Data are presented as mean ± SEM. Results are representative of three independent experiments. Source data are provided as a Source Data file.

Supplementary Figure 7

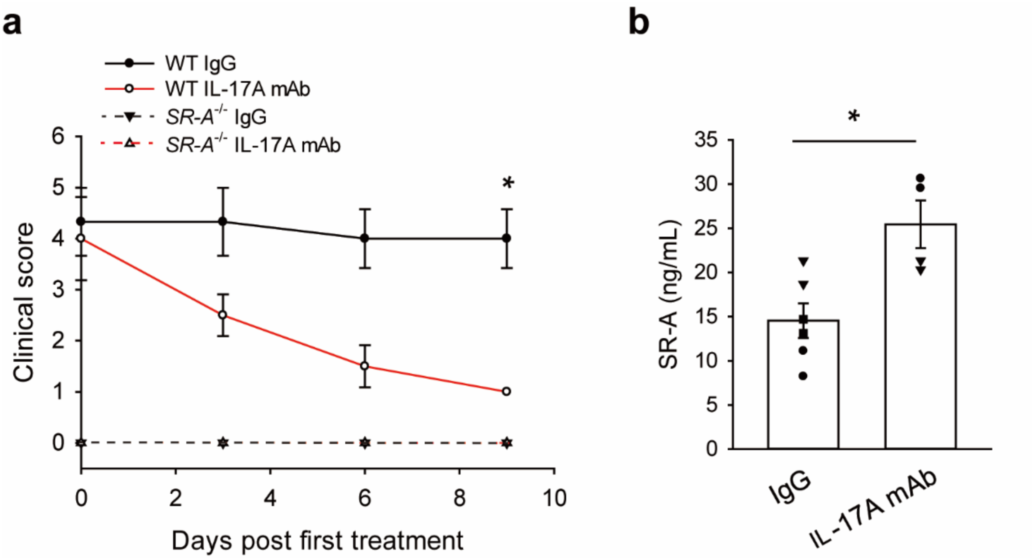

Supplementary Fig. 7 sSR-A production is not triggered by IL-17A in CIA mice. CIA was induced in WT and *SR-A*$^{-/-}$ mice. (**a**) Mice were treated with control IgG (n = 3 per group) or anti-IL-17A antibody (n = 2 or 3 per group) (200 μg, i.p.) once every three days starting from 28 days after induction. Disease progression was followed (**p* = 0.0276 at day 9). (**b**) sSR-A in serum of WT mice was detected by ELISA in duplicate 9 days after first anti-IL-17A antibody injection (**p* = 0.0100). Data are presented as mean ± SEM. Results are representative of three independent experiments. **p* < 0.05 (two-tailed Student's *t* test (**a**, **b**)).

Supplementary Figure 8

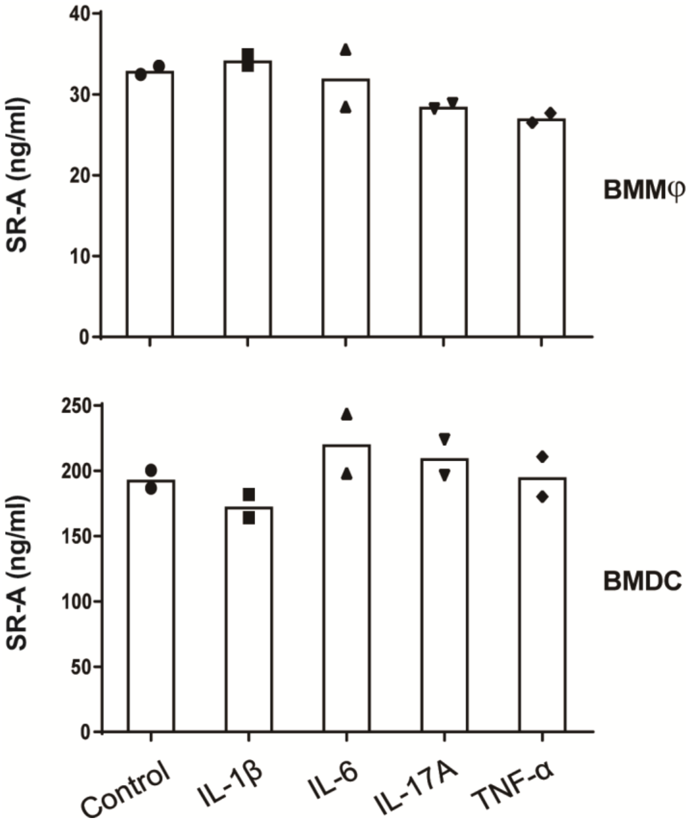

Supplementary Fig. 8 sSR-A production is not triggered by inflammatory cytokines. Bone marrow-derived macrophages (BMMφ) or bone marrow-derived dendritic cells (BMDC) from C57BL/6 mice were cultured in the presence of indicated cytokines (20 ng/mL) for 72 h. sSR-A in the culture medium was detected by ELISA. Results are representative of three independent experiments. Source data are provided as a Source Data file.

Supplementary Figure 9

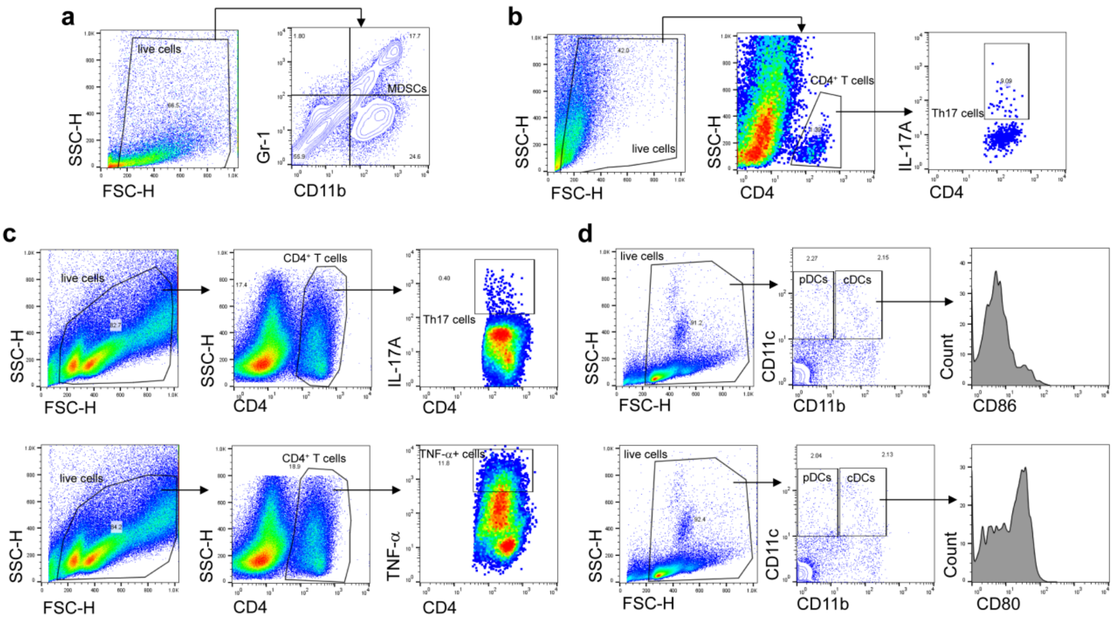

Supplementary Fig. 9 Gating strategies used for analyzing immune cell subsets, infiltration and activation in CIA mice. Gating strategies used for determination of the frequency of MDSCs (**a**, CD11b^{+}Gr-1^{+}) and Th17 cells (**b**, CD4^{+}IL-17A^{+}) as presented in Fig. 7d. (**c**) Gating strategies used for detection of Th17 cells (CD4^{+}IL-17A^{+}) and TNF-α producing CD4^{+} T cells as presented in Fig. 6h and Fig. 7g. (**d**) Gating strategies used for analyzing frequency of cDCs (conventional dendritic cells, CD11b^{+}CD11c^{+}) and pDCs (plasmacytoid dendritic cells, CD11b^{-}CD11c^{+}) as well as CD86/80 expression on cDCs in draining lymph nodes as presented in Supplementary Fig. 5a-b.

Supplementary Table 1

Comparison of clinical and immunological features between sSR-A-positive and sSR-A-negative RA patients.

Characteristics	Beijing cohort			Inner Mongolia cohort			Hangzhou cohort		
	sSR-A-positive	sSR-A-negative	*p*	sSR-A-positive	sSR-A-negative	*p*	sSR-A-positive	sSR-A-negative	*p*
Ages, year	59 (18-88)	58 (20-89)	0.425	59 (22-83)	60 (29-83)	0.479	60 (34-80)	57 (44-82)	0.647
Disease duration, year	10 (0.10-50)	8 (0.10-60)	0.046*	9 (0.04-40)	7.5 (0.08-40)	0.462	8 (0.10-50)	6 (0.30-30)	0.507
ESR, mm/h	48 (2-137)	36 (2-140)	0.014*	59.5 (3-125)	32 (3-122)	0.012*	65 (4-140)	46 (8-135)	0.051
CRP, mg/L	22.20 (1-741)	17.60 (0.21-381)	0.18	18.45 (0.60-181)	11.85 (3.16-126)	0.811	28.30 (1.14-190.70)	13.90 (0.23-154)	0.267
DAS28 (ESR)	4.96 (0.99-8.40)	4.60 (0.99-8.21)	0.096	5.90 (1.63-8.33)	4.50 (0.99-7.96)	0.002**	5.28 (2.78-8.57)	5.21 (2.26-8.67)	0.533
IgA, g/L	3.11 (0.46-36.20)	2.78 (0.49-9.08)	0.032*	2.72 (0.87-10)	2.33 (0.41-7.40)	0.006**	2.77 (1.33-8.26)	2.44 (1.31-6.75)	0.08
**IgM, g/L**	**1.27 (0.19-16.50)**	**1.01 (0.25-4.08)**	**0.000013*** **	**1.21 (0.19-28.50)**	**1.03 (0.20-2.06)**	**0.01***	**1.3 (0.39-4.22)**	**1.1 (0.38-3.48)**	**0.55**

IgG, g/L	13.30 (4.80-30.00)	13.70 (2.84-28.50)	0.799	13.00 (4.69-34.30)	11.80 (6.55-21.50)	0.046*	13.5 (3.06-29.70)	11.7 (4.95-21.50)	0.049*
**RF, IU/mL**	**212 (0.25-13800)**	**20 (0.21-1300)**	**5.5621E-21*** **	**240.5 (9.19-5110)**	**23.05 (9.19-4114)**	**1.6242E-7*** **	**177 (0-4610)**	**32.2 (0-2780)**	**1.89E-04*** **
Anti-CCP, U/ml	230.22 (2.08-387.18)	175.30 (2.08-525)	2.00E-06***	97 (5-200)	62.50 (5-200)	0.276	188.85 (0-1600)	142.1 (0-1600)	0.415
WBC, 10^9/L	6.60 (0.89-104)	6.12 (2.90-41.50)	0.072*	6.10 (2.20-12.30)	6.45 (2.50-10.10)	0.938	-	-	-
**GPI**	**2.05 (0.00-16.00)**	**0 (0-16.00)**	**1.5422E-15*** **	-	-	-	-	-	-

ESR: erythrocyte sedimentation rate; CRP: C-reactive protein; DAS28: disease activity score 28; Ig: immunoglobulin; RF: rheumatoid factor; Anti-CCP: anti-cyclic citrullinated peptide antibody; WBC: white blood cell; GPI: glucose-6-phosphate isomerase. The important items with significant difference are indicated by bold. The data are all in non-normal distribution, and are presented as median (range). **p* < 0.05, ***p* < 0.01, ****p* < 0.001, with exact *p* values shown in the table (two-tailed Mann-Whitney U test).

Supplementary Table 2

Associations between sSR-A and clinical/immunological features of RA patients after therapy (non-responders *vs.* responders).

Characteristics	Non-responders (n = 100)		Responders (n = 54)	
	r	*p*	*r*	*p*
Ages	-0.173	0.085	0.133	0.332
Disease duration	0.216	0.031*	0.200	0.143
ESR	0.197	0.050	0.076	0.580
CRP	0.162	0.107	0.022	0.883
IgA	-0.102	0.315	0.005	0.970
**IgM**	**0.443**	**0.000004*** **	0.070	0.621
IgG	-0.097	0.340	-0.006	0.966
**RF**	**0.612**	**5.372E-9*** **	0.094	0.573
Anti-CCP	0.307	0.002**	0.072	0.619
Albumin	-0.204	0.041*	-0.177	0.262
WBC	0.074	0.464	-0.082	0.592
**GPI**	**0.649**	**0.000059*** **	0.367	0.134
AKA	-0.83	0.602	-0.220	0.491
APF	0.126	0.380	-0.209	0.363

ESR: erythrocyte sedimentation rate; CRP: C-reactive protein; Ig: immunoglobulin; RF: rheumatoid factor; Anti-CCP: anti-cyclic citrullinated peptide antibody; WBC: white blood cell; GPI: glucose-6-phosphate isomerase; AKA: antikeratin antibodies; APF: antiperinuclear factor antibodies. The important items with significant association are indicated by bold. *$p < 0.05$, **$p < 0.01$, ***$p < 0.001$, with exact p values shown in the table (two-tailed Spearman's rank correlation test).

Supplementary Table 3

Clinical and demographic characteristics of RA patients in the diagnostic study.

Characteristics	Beijing cohort (n = 528)	Inner Mongolia cohort (n = 213)	Hangzhou cohort (n = 155)
Age, median (range), yrs	58 (18-89)	59 (22-83)	60 (34-82)
Sex, female/male	399/129	157/56	121/34
Disease duration, median (range), yrs	10 (0.1-60)	9 (0.04-40)	7 (0.1-50)
ESR, median (range), mm/h	43 (2-140)	53 (3-125)	57 (4-140)
CRP, median (range), mg/L	20.2 (0.21-741)	16.8 (0.6-181)	22.6 (0.23-190.7)
Tender joint count of 28 joints, median (range)	5 (0-28)	11 (0-28)	7.5 (0-28)
Swollen joint count of 28 joints, median (range)	4 (0-28)	2 (0-28)	2 (0-28)
DAS28, median (range)	4.9 (0.99-8.40)	5.55 (0.99-8.33)	5.25 (2.26-8.67)
Medication, no. (%)			
Steroids	193 (36.55)	128 (60.09)	99 (63.87)
NSAIDs	274 (51.89)	39 (18.31)	55 (35.48)
Methotrexate	216 (40.91)	103 (48.36)	64 (41.29)
Other DMARDs	498 (94.32)	165 (77.46)	140 (90.32)
Biologics	56 (10.61)	15 (7.04)	7 (4.52)
No treatment	4 (0.76)	0 (0)	1 (0.65)

896 RA patients were recruited overall (528 in Beijing cohort, 213 in Inner Mongolia cohort, and 155 in Hangzhou cohort). ESR: erythrocyte sedimentation rate; CRP: C-reactive protein; DAS28: disease activity score 28.

Supplementary Table 4

Clinical and demographic characteristics of SLE patients in the diagnostic study.

Characteristics	Beijing cohort (n = 254)	Inner Mongolia cohort (n = 144)	Hangzhou cohort (n = 80)
Age, median (range), yrs	36 (13-80)	43 (12-72)	34 (15-68)
Sex, no. female/male	232/22	135/9	77/3
Duration, median (range), yrs	5 (0.02-50)	7 (0.08-30)	5 (0.02-20)
ESR, median (range), mm/hr	28 (1-140)	37 (2-108)	29 (2-126)
CRP, median (range), mg/l	4.49 (1-183)	3.3 (0.62-118)	1.56 (0.1-126.9)
C3, mean (range), g/l	0.64 (0.09-1.68)	0.94 (0.19-1.86)	0.57 (0.22-1.27)
C4, median (range), g/l	0.12 (0.02-0.54)	0.16 (0.02-0.57)	0.07 (0.02-0.26)
Anti-dsDNA, no. positive/no. negative/no. nd	176/74/4	63/19/62	43/37/0
ANA, no. positive/no. negative/no. nd	231/16/7	132/6/6	70/10/0
AnuA, no. positive/no. negative/no. nd	144/98/12	——	35/45/0

478 SLE patients were recruited overall (254 in Beijing cohort, 144 in Inner Mongolia cohort, and 80 in Hangzhou cohort). ESR, erythrocyte sedimentation rate; CRP, C-reactive protein; C3, complement 3; C4, complement 4; Anti-dsDNA, anti-double-stranded DNA antibodies; ANA, antinuclear antibodies; AnuA, anti-nucleosome antibodies.

Supplementary Table 5

Clinical and demographic characteristics of SS patients in the diagnostic study.

Characteristics	Beijing cohort (n = 120)	Inner Mongolia cohort (n = 144)	Hangzhou cohort (n = 55)
Age, mean (range), yrs	57.37 (24-80)	54.27 (25-82)	52.75 (20-83)
Sex, no. female/male	118/2	139/5	52/3
Duration, median (range), yrs	8 (0.08-40)	4 (0.02-40)	3 (0.02-30)
Xerostomia, no. positive/no. negative	103/17	138/6	40/15
Xerophthalmia, no. positive/no. negative	92/28	107/37	32/23
Parotid enlargement, no. positive/no. negative	23/97	6/138	5/50
ESR, median (range), mm/hr	32.5 (3-140)	40 (2-119)	33 (3-123)
CRP, median (range), mg/L	3.61 (1-127)	3.31 (0.49-173)	0.86 (0.1-63.1)
Anti-SSA, no. positive/no. negative/no. nd	92/27/1	94/40/10	48/7/0
Anti-SSB, no. positive/no. negative/no. nd	40/79/1	31/95/18	34/21/0

319 SS patients were recruited overall (120 in Beijing cohort, 144 in Inner Mongolia cohort, and 55 in Hangzhou cohort). ESR, erythrocyte sedimentation rate; CRP, C-reactive protein; Anti-SSA, anti-SSA/Ro antibodies; Anti-SSB, anti-SSB/La antibodies.

Supplementary Table 6

Clinical and demographic characteristics of early RA (ERA) patients in the study.

Characteristics	ERA (≤ 24 m) (n = 251)	ERA (≤ 12 m) (n = 178)	ERA (≤ 6 m) (n = 91)
Age, mean (range), yrs	58.86 (23-88)	59.46 (23-88)	60.25 (23-88)
Sex, no. female/male	179/72	120/58	61/30
Duration, median (range), yrs	1 (0.04-2)	0.5 (0.04-1)	0.3 (0.04-0.5)
ESR, median (range), mm/hr	43 (3-135)	44 (3-135)	46.5 (4-135)
CRP, median (range), mg/L	19.5 (0.3-190.7)	21.4 (0.3-190.7)	26.8 (0.5-158)
Tender joint count, median (range) of 28 joints	6 (0-28)	6 (0-28)	6 (0-28)
Swollen joint count, median (range) of 28 joints	4 (0-28)	4 (0-28)	6.5 (0-28)
DAS28, mean (range)	5.06 (0.99-8.34)	5.12 (1.21-8.34)	5.19 (1.38-8.15)
Anti-CCP, no. positive/no. negative/no. nd	185/50/16	129/40/9	59/26/6
RF, no. positive/no. negative/no. nd	171/73/7	119/52/7	56/31/4
Medication, no. (%)			
Steroids	116 (46.2%)	85 (47.8%)	43 (47.3%)
NSAIDs	114 (45.4%)	89 (50%)	47 (51.6%)
Methotrexate	71 (28.3%)	36 (20.2%)	26 (28.6%)
Other DMARDs	193 (76.9%)	134 (75.3%)	72 (79.1%)
Biologics	27 (10.8%)	16 (9.0%)	8 (8.8%)
No treatment	0 (0%)	0 (0%)	0 (0%)

ESR, erythrocyte sedimentation rate; CRP, C-reactive protein; DAS28: disease activity score 28; Anti-CCP: anti-cyclic citrullinated peptide antibody; RF: rheumatoid factor.

Supplementary Table 7

Clinical and demographic characteristics of anti-CCP and/or RF negative RA patients in the study.

Characteristics	anti-CCP$^-$ RA (n = 179)	RF$^-$ RA (n = 276)	anti-CCP$^-$RF$^-$ RA (n = 155)
Age, median (range), years	59 (18-89)	59 (18-86)	59 (18-86)
Sex, no. female/male	132/47	214/62	122/33
Disease duration, median (range), years	7 (0.04-50)	7 (0.04-60)	6 (0.04-50)
ESR, median (range), mm/h	34.5 (2-122)	25 (2-200)	28 (2-122)
CRP, median (range), mg/L	14.1 (0.2-243)	10.6 (0.2-190.7)	9.07 (0.2-243)
Tender joint count, median (range) of 28 joints	8 (0-28)	3 (0-28)	9 (0-28)
Swollen joint count, median (range) of 28 joints	2 (0-28)	2 (0-28)	4 (0-28)
DAS28, median (range)	5.11 (0.68-8.09)	4.31 (0.68-8.37)	4.94 (1.38-8.01)
Medication, no. (%)			
Steroids	75 (41.90)	103 (37.32)	50 (32.26)
NSAIDs	62 (34.64)	99 (35.87)	43 (27.74)
Methotrexate	75 (41.90)	103 (37.32)	52 (33.55)
Other DMARDs	132 (73.74)	218 (78.99)	100 (64.52)
Biologics	9 (5.03)	15 (5.43)	8 (5.16)
No treatment	5 (2.79)	7 (2.54)	1 (0.65)

Anti-CCP: anti-cyclic citrullinated peptide antibody; RF, rheumatoid factor; ESR, erythrocyte sedimentation rate; CRP, C-reactive protein; DAS28: disease activity score 28.

Supplementary Table 8

Clinical and demographic characteristics of undifferentiated arthritis (UA) patients in the study.

Characteristics	undifferentiated arthritis (n = 119)
Age, median (range), yrs	50 (18-80)
Sex, female/male	78/41
Disease duration, median (range), yrs	1.17 (0.02-27)
ESR, median (range), mm/h	19 (2-118)
CRP, median (range), mg/L	6.2 (0.20-162)
Tender joint count, median (range) of 28 joints	3 (0-26)
Swollen joint count, median (range) of 28 joints	2 (0-26)
RF, no. positive/no. negative/no. nd	11/101/7
Anti-CCP, no. positive/no. negative/no. nd	16/95/8
Medication, no. (%)	
NSAIDs	71 (59.66%)
Methotrexate (MTX)	9 (7.56%)
Salazosulfapyridine (SASP)	15 (12.61%)
Hydroxychloroquine (HCQ)	20 (16.81%)
Other DMARDs	37 (31.09%)
Steroids	11 (9.24%)
No treatment	16 (13.45%)

ESR: erythrocyte sedimentation rate; CRP: C-reactive protein; RF: rheumatoid factor; Anti-CCP: anti-cyclic citrullinated peptide antibody.
